# Investigation of associations between retinal microvascular parameters and albuminuria in UK Biobank: a cross-sectional case-control study

**DOI:** 10.1186/s12882-021-02273-6

**Published:** 2021-02-25

**Authors:** Euan N. Paterson, Chris Cardwell, Thomas J. MacGillivray, Emanuele Trucco, Alexander S. Doney, Paul Foster, Alexander P. Maxwell, Gareth J. McKay, on behalf of The UK Biobank Eye and Vision Consortium, Tariq Aslam, Sarah Barman, Jenny Barrett, Paul Bishop, Peter Blows, Catey Bunce, Roxana Carare, Usha Chakravarthy, Michelle Chan, Antonietta Chianca, Valentina Cipriani, David Crabb, Philippa Cumberland, Alexander Day, Parul Desai, Bal Dhillon, Andrew Dick, Cathy Egan, Sarah Ennis, Paul Foster, Marcus Fruttiger, John Gallacher, David ( Ted) Garway-Heath, Jane Gibson, Dan Gore, Jeremy Guggenheim, Chris Hammond, Alison Hardcastle, Simon Harding, Ruth Hogg, Pirro Hysi, Pearse A. Keane, Sir Peng Tee Khaw, Anthony Khawaja, Gerassimos Lascaratos, Andrew Lotery, Phil Luthert, Tom MacGillivray, Sarah Mackie, Keith Martin, Bernadette McGuinness, Gareth McKay, Martin McKibbin, Danny Mitry, Tony Moore, James Morgan, Zaynah Muthy, Eoin O’Sullivan, Chris Owen, Praveen Patel, Euan Paterson, Tunde Peto, Axel Petzold, Jugnoo Rahi, Alicja Rudnicka, Jay Self, Sobha Sivaprasad, David Steel, Irene Stratton, Nicholas Strouthidis, Cathie Sudlow, Caroline Thaung, Dhanes Thomas, Emanuele Trucco, Adnan Tufail, Marta Ugarte, Veronique Vitart, Stephen Vernon, Ananth Viswanathan, Cathy Williams, Katie Williams, Jayne Woodside, Max Yates, Jennifer Yip, Yalin Zheng, Haogang Zhu, Robyn Tapp, Denize Atan, Alexander Doney

**Affiliations:** 1 Centre for Public Health, Institute of Clinical Science, Queen’s University Belfast, Block B, Royal Hospital, Grosvenor Road, Belfast, Northern Ireland BT12 6BA; 2 VAMPIRE project, Centre for Clinical Brain Sciences, University of Edinburgh, Edinburgh, Scotland UK; 3 VAMPIRE project, Computer Vision and Image Processing Group, School of Science and Engineering (Computing), University of Dundee, Dundee, UK; 4 Ninewells Hospital and Medical School, University of Dundee, Dundee, UK; 5 Moorfields Eye Hospital, London, UK

## Abstract

**Background:**

Associations between microvascular variation and chronic kidney disease (CKD) have been reported previously. Non-invasive retinal fundus imaging enables evaluation of the microvascular network and may offer insight to systemic risk associated with CKD.

**Methods:**

Retinal microvascular parameters (fractal dimension [FD] – a measure of the complexity of the vascular network, tortuosity, and retinal arteriolar and venular calibre) were quantified from macula-centred fundus images using the Vessel Assessment and Measurement Platform for Images of the REtina (VAMPIRE) version 3.1 (VAMPIRE group, Universities of Dundee and Edinburgh, Scotland) and assessed for associations with renal damage in a case-control study nested within the multi-centre UK Biobank cohort study. Participants were designated cases or controls based on urinary albumin to creatinine ratio (ACR) thresholds. Participants with ACR ≥ 3 mg/mmol (ACR stages A2-A3) were characterised as cases, and those with an ACR < 3 mg/mmol (ACR stage A1) were categorised as controls. Participants were matched on age, sex and ethnic background.

**Results:**

Lower FD (less extensive microvascular branching) was associated with a small increase in odds of albuminuria independent of blood pressure, diabetes and other potential confounding variables (odds ratio [OR] 1.18, 95% confidence interval [CI] 1.03–1.34 for arterioles and OR 1.24, CI 1.05–1.47 for venules). Measures of tortuosity or retinal arteriolar and venular calibre were not significantly associated with ACR.

**Conclusions:**

This study supports previously reported associations between retinal microvascular FD and other metabolic disturbances affecting the systemic vasculature. The association between retinal microvascular FD and albuminuria, independent of diabetes and blood pressure, may represent a useful indicator of systemic vascular damage associated with albuminuria.

**Supplementary Information:**

The online version contains supplementary material available at 10.1186/s12882-021-02273-6.

## Background

The retina possesses several characteristics that represent potential biomarkers for conditions caused by, or resulting in, microvascular dysfunction, such as chronic kidney disease (CKD). Indeed, variation in retinal microvascular calibre has been reported in association with a range of vascular conditions such as coronary heart disease [1], hypertension [2], stroke [3], and diabetes [4], highlighting the potential of retinal microvascular parameters (RMP) for screening systemic microvascular variation associated with CKD.

The retina has a higher metabolic demand than most other tissues [5, 6] yet its inner layers are supplied blood by a particularly sparse microvascular network [6]. As such, the retinal microvasculature may exhibit particularly responsive structural and physiological adaptations to reduced blood supply. Furthermore, the microvasculature supplying the inner retinal layers can be imaged non-invasively using widely available fundus cameras, allowing the quantification of geometric measures such as vessel calibre, fractal dimension and tortuosity.

Fractal dimensions (FD) provide a quantification of the complexity of the vascular network model related to the iterative growth and branching processes involved in the formation of the vascular bed. Deviation from ‘normal’ FD and other RMPs may indicate vascular growth patterns associated with certain pathologies [7]. An optimal vascular geometry provides oxygen and nutrients to cells with minimal energy expenditure and deviations from healthy microvascular geometry may result from, or represent increased susceptibility to, CKD-related vascular impairment [8]. Factors influencing FD, such as impaired vessel growth, vessel rarefaction and dropout, have been reported in association with atherosclerosis, hypertension, Alzheimer’s disease, CKD and diabetes [9]. Retinal microvascular variation in characteristics such as FD and vessel tortuosity, may reflect systemic changes associated with cardiovascular disease (CVD) [10, 11], diabetes [12], and hypertension [10, 13]. Similarly, changes in vessel tortuosity have also been reported in large and small vessels throughout the body in imaging studies of atherosclerosis, hypertension and diabetes [14].

In this study, a nested case-control comparison used participant data from the UK Biobank study (UKBB) [15, 16] to assess associations between retinal microvascular FD, tortuosity and calibre and urinary albumin creatinine ratio (ACR). The UKBB study is a large, UK-based, cohort study of middle-aged and older adults undertaken to improve the prevention, diagnosis and treatment of a wide range of serious and life-threatening illnesses. The UKBB study collected data on a large number of variables including urinary albumin and creatinine and digital fundus photographs in a subset of participants. Associations between a range of RMP and renal damage were evaluated in a nested subset of the UKBB population.

## Methods

### Study design and data collection

A cross-sectional, nested case-control study was performed using data from the UKBB population and data collection has been described previously [15, 16]. Briefly, UKBB is a multi-centre cohort study that recruited 502,616 people aged between 40 and 69 years, from the UK population. Baseline data collection was carried out between 2006 and 2010. Participants attended assessment centre visits where interviews were conducted, including touchscreen and web-based dietary questionnaires, and measurements were taken of blood pressure and physical measures including height, weight and waist circumference; blood and urine samples were collected. Data relating to eye health (visual acuity and intraocular pressure etc.) were collected on ~ 112,000 participants. Digital fundus photographs were available for 67,308 participants. Eye imaging was undertaken at baseline assessment centre visits between December 2009 and July 2010. Data collection and analysis was performed according to the principles of the Declaration of Helsinki under ethical approval from the North West Multi-Centre Research Ethics committee (06/MRE08/65).

### Participant eligibility

Participants were eligible for inclusion in the present study if eye images were available in addition to urinary albumin, urinary creatinine, age, sex and ethnic background data.

### Case-control criteria

Participants were selected as cases and controls based on clinical ACR thresholds calculated from urinary albumin and creatinine measurements taken at baseline assessment centre visits. Participants with ACR ≥ 3 mg/mmol (ACR stages A2-A3) were categorised as cases, and those with an ACR < 3 mg/mmol (ACR stage A1) as controls.

### Participant selection

**Fig. 1:**
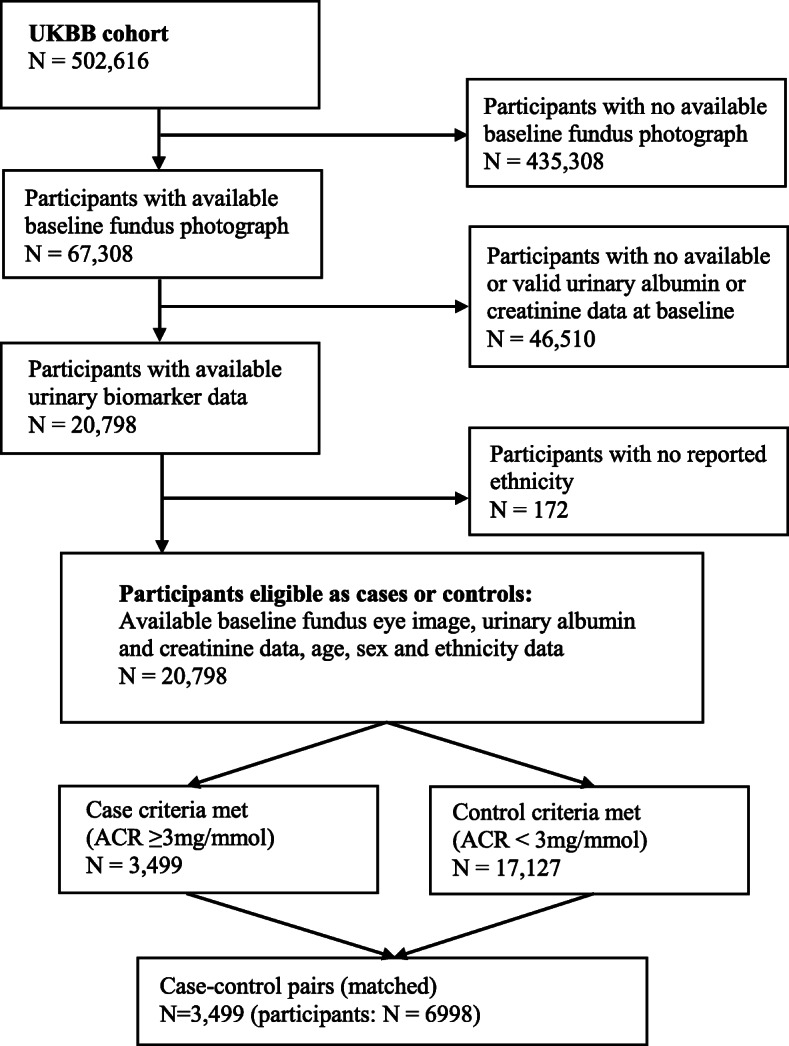
Study cohort eligibility selection criteria

Of the 67,308 UKBB participants with retinal images, 3499 had ACR ≥ 3 mg/mmol, meeting the inclusion criteria and case designation. A further 20,626 participants with available data and ACR < 3 mg/mmol were eligible controls (for a flow diagram of the derivation of the study cohort see Fig. 1) and 3499 age and sex-matched controls were included in the analyses. Participants were randomly individually matched without replacement based on sex and ethnic background categories (White; Asian or Asian British; Black or Black British; Mixed/Chinese/Other) with age (age at recruitment) of cases matched as closely as possible (age differences up 2 years were tolerated). Of the 6998 participants included, 1411 (20%) did not have right eye images of sufficient quality for VAMPIRE image analysis. Of the remaining 5587 (80%) participants, RMP based on the four largest vessels (including the retinal arcades) were derived for 1890 participants, consisting of 920 cases and 970 controls.

### Estimation of glomerular filtration rate (eGFR)

eGFR was calculated using the CKD-EPI equations [17] based on single serum creatinine (SCr) and cystatin C (Cys) measurements taken during baseline assessment centre visits.

### Image capture and retinal vessel assessment

Macula-centred digital retinal fundus images were captured using a Topcon 3D OCT 1000 Mark 2 camera (Topcon Great Britain Medical Ltd., Berkshire, UK) at baseline visit. RMP were calculated from fundus images using the Vessel Assessment and Measurement Platform for Images of the REtina (VAMPIRE) version 3.1 (VAMPIRE group, Universities of Dundee and Edinburgh, Scotland). RMP included the central retinal arteriolar equivalent (CRAE), central retinal venular equivalent (CRVE), the arteriovenous ratio (AVR), retinal vascular FD and tortuosity. Trained graders performed image analysis and were blinded to participant characteristics. Inter-grader reliability was assessed to ensure consistency of measurements between graders using intra-class correlation coefficients (ICCs). ICCs were calculated at intervals and the mean ICCs for this study were 0.92 (CRAE) and 0.93 (CRVE), above the thresholds considered “excellent” [18, 19].

### Statistical analyses

Statistical analyses were performed using Stata/1C version 14.2 (Timberlake Consultants Limited, Richmond upon Thames, UK). The central tendency and spread of continuous variables were summarised using the mean and standard deviation (SD) respectively. Categorical variables were summarised using the frequency and percentage. Between-group comparisons were performed using t-tests for continuous variables and Chi squared tests for categorical variables.

Logistic regression models were used to test associations between each RMP (as independent variables) and ACR category, and, for secondary analyses, prevalence of eGFR < 60 ml/min/1.73m^2^ and prevalence of either ACR > 3 mg/mmol and/or eGFR < 60 ml/min/1.73m^2^. RMP were assessed as z-scores where a single unit change represented one standard deviation decrease in the RMP. Three models were considered for each RMP: Model 1 was unadjusted; Model 2 was adjusted for age, sex, waist circumference, systolic blood pressure, blood pressure-lowering medication usage, presence of diabetes mellitus, smoking history (as a binary variable, ever smoked versus never smoked), ethnicity (as a binary variable, white ethnicity versus non-white ethnicity), and alcohol consumption (ever versus never). Model 3 included variables in model 2, in addition to visual acuity (assessed using a logarithm of the minimal angle of resolution [logMAR] chart), cornea corrected intraocular pressure (IOP), and a history of eye surgery (as a binary variable), to account for the potential effects of magnification and retinal abnormalities. Sensitivity analyses excluded participants with eGFR < 60 ml/min/1.73m^2^ and additional adjustment for a history of stroke, heart attack and angina.

### Power and sample size calculations

Sample size and power calculations suggested that a study including 600 cases and 600 controls would have > 90% power to detect an association between retinal vessel calibre and CKD of similar magnitude to the association reported by Sabanayagam et al., 2009 [20] (an odds ratio [OR] of 1.68 for CKD) based on a test for trend in CKD risk across quarters of the distribution of retinal vessel calibre.

## Results

The summary characteristics for the 1877 participants (963 cases, and 914 controls) with RMP are shown in Table 1. The mean age of participants was 58 years (SD 8), mean waist circumference was 93.0 cm (SD 14.8), mean systolic blood pressure was 146 mmHg (SD 22), mean eGFR was 88.8 ml/min/1.73m^2^ (SD 16.0), 1033 (55%) were female, 347 (19%) used blood pressure-lowering medication, 209 (11%) had diabetes, 1098 (59%) had previously smoked, 1642 (88%) were identified as white ethnicity, and 1752 (93%) had previously consumed alcohol. Of the participants with ACR ≥ 3 mg/mmol (ACR stages A2-A3), 897 (98%) had moderately increased ACR between 3 and 30 mg/mmol (equivalent to ACR stage A2), and 17 (2%) had severely increased ACR > 30 mg/mmol (equivalent to ACR stage A3). Compared to those with ACR stage A1, those with ACR stages 2–3 had higher systolic blood pressure (143 mmHg (SD 21) vs 150 mmHg (SD 22), *p* < 0.001), and intraocular pressure (IOP: 16.41 mmHg (SD 4.78) vs 16.54 mmHg (SD 4.12), *p* < 0.001), more prevalent use of blood pressure lowering medication (16% vs 21%, *p* < 0.001), prevalent diabetes (8% vs 14%, *p* < 0.001) and lower retinal arteriolar (1.28 (SD 0.11) vs 1.27 (SD 0.10), *p* = 0.01) and venular fractal dimension (1.32 (SD 0.10) vs 1.3 (SD 0.10), *p* = 0.01). There were no significant differences in eGFR, retinal microvascular calibre (CRAE, CRVE and AVR) or tortuosity between cases and controls. Comparisons of population characteristics for all participants that met the inclusion criteria compared to those in which RMPs were generated, is presented in Table S1.

**Table 1:** Population summary characteristics

	All (*n* = 1877)	ACR (*n* = 963) < 3 mg/mmol	ACR (*n* = 914) ≥ 3 mg/mmol	*p*
	Mean (SD)	Mean (SD)	Mean (SD)	
Age (years)	58 (8)	58 (8)	58 (8)	0.58
Waist circumference (cm)	93 (15)	93 (15)	93 (15)	0.53
Systolic blood pressure (mmHg)	146 (22)	143 (21)	150 (22)	< 0.001
eGFR (ml/min/1.73m^2^)	88.8 (16.0)	88.5 (14.5)	89.1 (17.4)	0.39
IOP (mmHg)	16.48 (4.48)	16.41 (4.78)	16.54 (4.12)	< 0.001
LogMAR	0.03 (0.20)	0.03 (0.19)	0.04 (0.20)	0.07
CRAE (px)	22.35 (1.97)	22.33 (2.01)	22.38 (1.93)	0.62
CRVE (px)	28.70 (2.90)	28.71 (2.77)	28.68 (3.02)	0.87
AVR	0.78 (0.10)	0.78 (0.09)	0.78 (0.11)	0.19
FDa	1.28 (0.10)	1.28 (0.11)	1.27 (0.10)	0.01
FDv	1.31 (0.10)	1.32 (0.10)	1.30 (0.10)	0.01
Torta	3.00^+^ (9.00^+^)	3.00^+^ (9.00^+^)	3.00^+^ (9.00^+^)	0.78
Tortv	2.00^+^ (4.00^+^)	2.00^+^ (5.00^+^)	2.00^+^ (4.00^+^)	0.36
	Number (%)	Number (%)	Number (%)	*p*
ACR stage A2	897 (48)	0 (0)	897 (98)	< 0.001
ACR stage A3	17 (1)	0 (0)	17 (2)	< 0.001
eGFR < 60 ml/min/1.73m^2^	65 (4)	21 (2)	44 (5)	0.001
Sex (Female)	1033 (55)	521 (54)	512 (56)	0.4
Blood pressure-lowering medication usage	347 (19)	152 (16)	195 (21)	< 0.001
Diabetes mellitus	209 (11)	78 (8)	131 (14)	< 0.001
Ever smoked	1098 (59)	543 (57)	555 (61)	0.06
White ethnicity	1642 (88)	846 (88)	796 (87)	0.58
Ever consumed alcohol	1752 (93)	905 (94)	847 (93)	0.22
Ever eye surgery	127 (7)	63 (7)	64 (7)	0.69

*SD* standard deviation, *eGFR* Estimated glomerular filtration rate, *IOP* Intraocular pressure, *LogMAR* visual acuity assessed using a Logarithm of the Minimum Angle of Resolution chart, *CRAE* central retina arteriolar equivalent, *CRVE* central retinal venular equivalent, *px* pixels, *AVR* arteriovenous ratio, *FDa/v* arteriolar/venular fractal dimension, *Torta/v* arteriolar/venular tortuosity

### Associations between retinal microvascular parameters and albuminuria categories

**Fig. 2:**
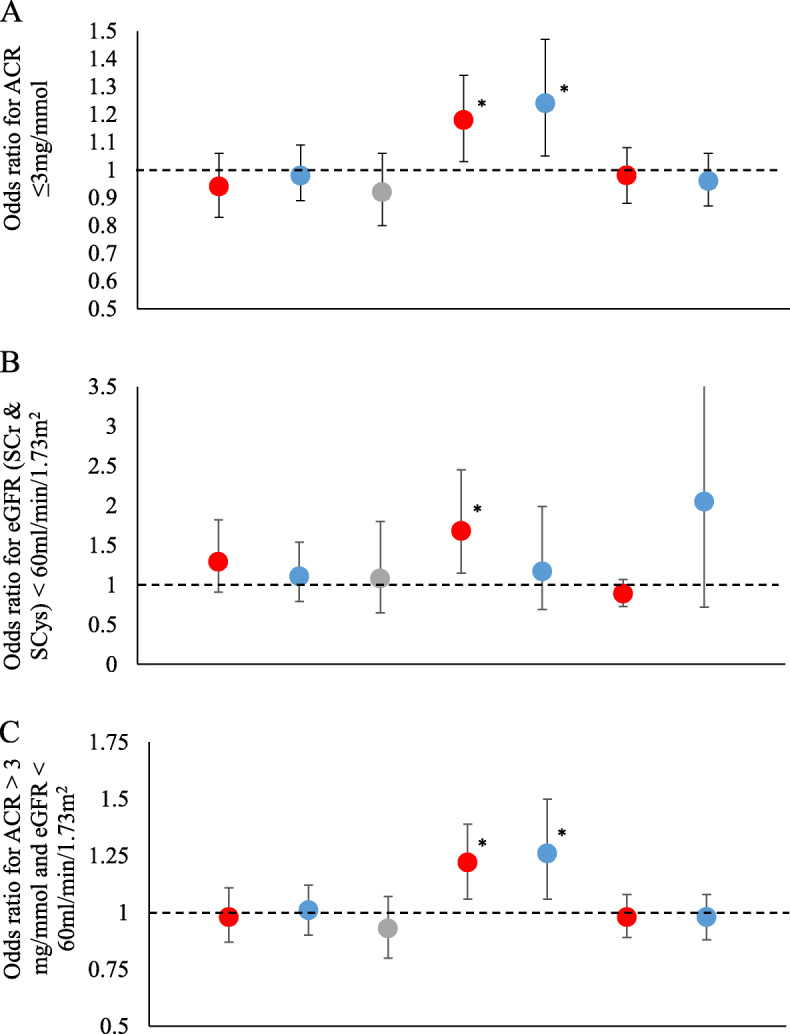
Associations between retinal microvascular parameters and measures of renal function and damage. Associations between retinal microvascular parameters (Z scores) and **a**: ACR > 3 mg/mmol; **b**: eGFR < 60 ml/min/1.73m^2^ (combined CKD-EPI for both serum creatinine and serum cystatin C); **c**: ACR > 3 mg/mmol and/or eGFR < 60 ml/min/1.73m^2^ combined CKD-EPI for both serum creatinine and serum cystatin C), for Model 2 (adjusted for age, sex, waist circumference, systolic blood pressure, blood pressure-lowering medication usage, presence of diabetes mellitus, smoking history (as a binary variable, ever smoked versus never smoked), ethnicity (as a binary variable, white ethnicity versus non-white ethnicity), and alcohol consumption (ever versus never)).*Association significant at the *p* < 0.05 level. ACR: albumin to creatinine ratio; CRAE: central retina arteriolar equivalent; CRVE: central retinal venular equivalent; AVR: arteriovenous ratio; FDa/v: arteriolar/venular fractal dimension; Torta/v: arteriolar/venular tortuosity. Bars indicate 95% confidence intervals

Measures of arteriolar and venular calibre (CRAE, CRVE and AVR) were not significantly associated with ACR category (ACR stage A1 vs A2-A3) in unadjusted or adjusted models (Table 2). Decreased FD (both arteriolar [FDa] and venular fractal dimension [FDv]) was associated with greater odds of ACR stages A2-A3. In model 2, adjusted for age, sex, waist circumference, systolic blood pressure, blood pressure-lowering medication usage, presence of diabetes mellitus, smoking history, ethnicity, and alcohol consumption, a single standard deviation decrease in FDa and FDv were associated with an increased risk of albuminuria (OR = 1.16, 95% CI (1.02–1.32) and 1.25, 95% CI (1.06–1.48), respectively), Table 2, Fig. 2a. Additional adjustment for IOP, logMAR visual acuity, and a history of eye-surgery did not significantly alter the effect size or direction of associations observed. Microvascular tortuosity was not associated with albuminuria in any of the models tested. Additional adjustment for eGFR did not substantially alter the effect size of the observed association (results not shown). Similarly, in a sensitivity analysis, that further excluded control participants with eGFR < 60 ml/min/1.73m^2^ (CKD stages 3–5), did not significantly alter the effect size of the associations observed (results not shown).

**Table 2:** Change in odds associated with ACR stages A2-A3 (compared with stage A1) per standard deviation increase in retinal microvascular parameters

	Model 1	***p***	Model 2	***p***	Model 3	***p***
	OR (95% CI)		OR (95% CI)		OR (95% CI)	
CRAE	0.97 (0.87, 1.09)	0.62	0.93 (0.82, 1.04)	0.19	0.94 (0.83, 1.06)	0.31
CRVE	1.01 (0.91, 1.11)	0.87	0.97 (0.88, 1.08)	0.62	0.98 (0.89, 1.09)	0.77
AVR	0.92 (0.81, 1.04)	0.19	0.92 (0.80, 1.05)	0.23	0.92 (0.80, 1.06)	0.23
FDa	1.18 (1.04, 1.33)	0.01	1.16 (1.02, 1.32)	0.02	1.18 (1.03, 1.34)	0.02
FDv	1.24 (1.06, 1.45)	0.01	1.25 (1.06, 1.48)	0.01	1.24 (1.05, 1.47)	0.01
Torta	0.99 (0.90, 1.08)	0.78	0.99 (0.90, 1.09)	0.83	0.98 (0.88, 1.08)	0.62
Tortv	0.96 (0.86, 1.06)	0.37	0.96 (0.87, 1.07)	0.47	0.96 (0.87, 1.06)	0.43

Associations between retinal microvascular parameters (Z scores) vs ACR category. Model 1: unadjusted; Model 2: adjusted for age, sex, waist circumference, systolic blood pressure, blood pressure-lowering medication usage, presence of diabetes mellitus, smoking history (as a binary variable, ever smoked versus never smoked), ethnicity (as a binary variable, white ethnicity versus non-white ethnicity), and alcohol consumption (ever versus never); Model 3: adjustment for variables in model 2 and further adjusted for visual acuity (logMAR), cornea corrected intraocular pressure, and history of eye surgery (as a binary variable)

*OR* odds ratio, *CI* confidence interval, *CRAE* central retina arteriolar equivalent, *CRVE* central retinal venular equivalent, *AVR* arteriovenous ratio, *FDa/v* arteriolar/venular fractal dimension, *Torta/v* arteriolar/venular tortuosity

### Associations between retinal microvascular parameters and eGFR < 60 ml/min/1.73m^2^

Associations between RMP and eGFR < 60 ml/min/1.73m^2^ are shown for eGFR calculated using SCr (Table 3) and for eGFR calculated using both SCr & SCys (Table 4 and Fig. 2b). Measures of arteriolar and venular calibre (CRAE, CRVE and AVR) were not associated with eGFR (SCr) < 60 ml/min/1.73m^2^ in the unadjusted or adjusted model 2. Following adjustment for IOP, visual acuity and history of eye surgery, narrower retinal arterioles were significantly associated with increased odds of eGFR (SCr) < 60 ml/min/1.73m^2^ (OR = 1.63, 95% CI: 1.15, 2.31). However, calculation of eGFR based on both SCr and SCys, identified no significant associations with arteriolar or venular calibre. Lower FDa was also significantly associated with eGFR < 60 ml/min/1.73m^2^ in all models, regardless of eGFR estimation approach. In fully adjusted models, a single standard deviation decrease in FDa was associated with and OR = 1.68 (1.15, 2.45) for eGFR (combined SCr and SCys) < 60 ml/min/1.73m^2^ (CKD stages 3–5), Table 4 and Fig. 2b. Microvascular tortuosity was not associated with CKD stages 3–5 in any of the models tested. Additional sensitivity analyses categorising participants by eGFR < 60 ml/min/1.73m^2^ based on the CKD-EPI equation for SCys only, showed little difference in effect to the findings reported for the combined SCr and SCys equation.

**Table 3:** Associations between retinal microvascular parameters (Z scores) vs eGFR (SCr) < 60 ml/min/1.73m^2^

	Model 1	***p***	Model 2	***p***	Model 3	***p***
	OR (95% CI)		OR (95% CI)		OR (95% CI)	
CRAE	1.30 (0.95, 1.78)	0.10	1.37 (0.99, 1.91)	0.06	1.63 (1.15, 2.31)	0.01
CRVE	1.25 (0.94, 1.67)	0.13	1.25 (0.91, 1.70)	0.17	1.24 (0.89, 1.75)	0.21
AVR	1.01 (0.67, 1.52)	0.98	1.07 (0.68, 1.70)	0.76	1.30 (0.79, 2.15)	0.31
FDa	1.50 (1.09, 2.08)	0.01	1.57 (1.11, 2.24)	0.01	1.57 (1.10, 2.24)	0.01
FDv	1.28 (0.82, 2.01)	0.28	1.20 (0.72, 1.99)	0.48	1.15 (0.68, 1.96)	0.60
Torta	0.93 (0.77, 1.12)	0.43	0.93 (0.73, 1.17)	0.53	0.90 (0.72, 1.12)	0.35
Tortv	1.85 (0.74, 4.62)	0.19	1.73 (0.72, 4.18)	0.22	1.65 (0.66, 4.15)	0.28

Associations between retinal microvascular parameters (Z scores) vs eGFR < 60 ml/min/1.73m^2^ based on serum creatinine. Model 1: unadjusted; Model 2: adjusted for age, sex, waist circumference, systolic blood pressure, blood pressure-lowering medication usage, presence of diabetes mellitus, smoking history (as a binary variable, ever smoked versus never smoked), ethnicity (as a binary variable, white ethnicity versus non-white ethnicity), and alcohol consumption (ever versus never); Model 3: adjustment for variables in model 2 and further adjusted for visual acuity (logMAR), cornea corrected intraocular pressure, and history of eye surgery (as a binary variable)

*OR* odds ratio, *CI* confidence interval, *CRAE* central retina arteriolar equivalent, *CRVE* central retinal venular equivalent, *AVR* arteriovenous ratio, *FDa/v* arteriolar/venular fractal dimension, *Torta/v* arteriolar/venular tortuosity

**Table 4:** Associations between retinal microvascular parameters (Z scores) vs eGFR (SCr & SCys) < 60 ml/min/1.73m^2^

	Model 1	***p***	Model 2	***p***	Model 3	***p***
	OR (95% CI)		OR (95% CI)		OR (95% CI)	
CRAE	1.20 (0.88, 1.62)	0.25	1.17 (0.84, 1.62)	0.36	1.29 (0.91, 1.82)	0.15
CRVE	1.10 (0.83, 1.47)	0.49	1.10 (0.81, 1.50)	0.55	1.11 (0.79, 1.54)	0.55
AVR	1.02 (0.68, 1.52)	0.94	0.98 (0.61, 1.57)	0.94	1.08 (0.65, 1.80)	0.77
FDa	1.50 (1.08, 2.09)	0.02	1.65 (1.13, 2.41)	0.01	1.68 (1.15, 2.45)	0.01
FDv	1.35 (0.87, 2.09)	0.18	1.27 (0.77, 2.09)	0.35	1.17 (0.69, 1.99)	0.55
Torta	0.90 (0.77, 1.05)	0.20	0.90 (0.74, 1.10)	0.29	0.89 (0.73, 1.07)	0.22
Tortv	1.94 (0.77, 4.88)	0.16	1.97 (0.75, 5.21)	0.17	2.05 (0.72, 5.83)	0.18

Associations between retinal microvascular parameters (Z scores) vs eGFR < 60 ml/min/1.73m^2^ based on serum creatinine and cystatin C. Model 1: unadjusted; Model 2: adjusted for age, sex, waist circumference, systolic blood pressure, blood pressure-lowering medication usage, presence of diabetes mellitus, smoking history (as a binary variable, ever smoked versus never smoked), ethnicity (as a binary variable, white ethnicity versus non-white ethnicity), and alcohol consumption (ever versus never); Model 3: adjustment for variables in model 2 and further adjusted for visual acuity (logMAR), cornea corrected intraocular pressure, and history of eye surgery (as a binary variable). OR: odds ratio; CI: confidence interval

### Associations between retinal microvascular parameters and either ACR > 3 mg/mmol or eGFR < 60 ml/min/1.73m^2^

Associations between RMP and ACR > 3 mg/mmol or eGFR < 60 ml/min/1.73m^2^ (SCr & SCys) are shown in Table 5. Measures of microvascular calibre (CRAE, CRVE and AVR) were not significantly associated with ACR > 3 mg/mmol or eGFR < 60 ml/min/1.73m^2^ in any of the models tested. A single SD decrease in FD (both FDa and FDv) was significantly associated with ACR > 3 mg/mmol or eGFR < 60 ml/min/1.73m^2^ in all models tested (Table 5, Fig. 2c). In the fully adjusted model, a single SD decrease in FDa and FDv was significantly associated with an increase in odds = 1.22, 95% CI (1.06–1.39) and 1.26, 95% CI (1.06–1.50) respectively. Microvascular tortuosity was not associated with ACR > 3 mg/mmol or eGFR < 60 ml/min/1.73m^2^ in any of the models tested. Further adjustment for stroke, heart attack and angina in a sensitivity analysis did not significantly alter the findings reported (data not shown).

**Table 5:** Associations between retinal microvascular parameters (Z scores) vs CKD status based on ACR > 3 mg/mmol and/or eGFR < 60 ml/min/1.73m^2^ (SCr & SCys)

	Model 1	***p***	Model 2	***p***	Model 3	***p***
	OR (95% CI)		OR (95% CI)		OR (95% CI)	
CRAE	1.01 (0.90, 1.13)	0.88	0.97 (0.86, 1.09)	0.59	0.98 (0.87, 1.11)	0.79
CRVE	1.03 (0.93, 1.14)	0.59	0.99 (0.89, 1.10)	0.90	1.01 (0.90, 1.12)	0.92
AVR	0.92 (0.81, 1.05)	0.21	0.93 (0.80, 1.06)	0.27	0.93 (0.80, 1.07)	0.29
FDa	1.22 (1.07, 1.39)	0.003	1.20 (1.05, 1.37)	0.01	1.22 (1.06, 1.39)	0.01
FDv	1.27 (1.08, 1.49)	0.004	1.28 (1.08, 1.52)	0.01	1.26 (1.06, 1.50)	0.01
Torta	0.99 (0.90, 1.09)	0.85	1.00 (0.90, 1.10)	0.92	0.98 (0.89, 1.08)	0.69
Tortv	0.97 (0.88, 1.07)	0.52	0.98 (0.88, 1.08)	0.65	0.98 (0.88, 1.08)	0.63

Associations between retinal microvascular parameters (Z scores) vs CKD status based on ACR > 3 mg/mmol and/or eGFR < 60 ml/min/1.73m^2^ (based on serum creatinine and serum cystatin C). Model 1: unadjusted; Model 2: adjusted for age, sex, waist circumference, systolic blood pressure, blood pressure-lowering medication usage, presence of diabetes mellitus, smoking history (as a binary variable, ever smoked versus never smoked), ethnicity (as a binary variable, white ethnicity versus non-white ethnicity), and alcohol consumption (ever versus never); Model 3: adjustment for variables in model 2 and further adjusted for visual acuity (logMAR), cornea corrected intraocular pressure, and history of eye surgery (as a binary variable)

*OR* odds ratio, *CI* confidence interval

## Discussion

In this nested case-control study of UKBB participants, we evaluated associations between RMP and albuminuria (ACR > 3 mg/mmol) and eGFR < 60 ml/min/1.73m^2^ in a secondary analysis. Reduced FD (lower geometric complexity of the vascular network) was associated with moderately increased odds of albuminuria independent of blood pressure, diabetes and other potential confounding variables. Given the previously reported associations between retinal microvascular FD and other metabolic and barometric disturbances affecting the systemic vasculature [10–12], and the associations identified in this study independent of diabetes and blood pressure, retinal microvascular FD may be a useful indicator of systemic vascular damage associated with albuminuria. Lower arteriolar FD was also associated with a moderately greater risk of eGFR < 60 ml/min/1.73m^2^. This study returned little evidence that measures of retinal microvascular calibre (CRAE, CRVE, and AVR) or tortuosity were associated with ACR.

Normal organ development manifests through optimal microvascular branching to maximise oxygen and nutrient supply with minimal energy expenditure [7, 8]. Given the microvascular damage that contributes to CKD [21], retinal imaging may provide opportunistic non-invasive assessment of the microvascular variation associated with CKD risk. However, CKD also results in systemic microvascular damage due to calcification [22, 23], altered blood pressure, and endothelial dysfunction [24–26]. Indeed, CKD is associated with an increased risk of overt microvascular changes in the retina, such as retinopathy [27, 28], although this association may arise from the effects of shared risk factors [29]. Therefore, there is an issue of reverse causality whereby FD may be associated with both the causes and effects of CKD. Furthermore, the possibility of retro-causality exists whereby, vascular effects of renal dysfunction may manifest early in the disease process [30–32], with sub-clinical reductions in renal function, which may also result in detectible variation in RMPs prior to clinical CKD diagnosis. Indeed, the retina is particularly sensitive to alterations in oxygen supply as a result of its high oxygen demand [33], and retinal microvascular fractals are also significantly altered in response to other conditions involving vascular and neuronal health [10–12, 21–23]. Therefore, FD may prove sufficiently sensitive to serve as an indicator of microvascular damage. Nevertheless, the accuracy of automated estimates of FD from retinal images remains a work in progress [34–36].

Few studies have previously assessed associations between ACR and retinal microvascular tortuosity or FD. In the Singapore Malay Eye Study, greater FD was associated with reduced likelihood (OR 0.76) of albuminuria in adults of Malay ethnicity [37]. Similarly, the present study found associations of similar strength and direction in a population of largely European descent. In addition, The Singapore Malay Eye Study also reported no significant associations between arteriolar or venular tortuosity and ACR [37].

Retinal microvascular calibre has been previously proposed as a potential biomarker for CKD from data generated from several population-based studies but the findings reported have been inconsistent. The findings of the present study support a previous lack of reported association between CRAE and ACR [37–40], in contrast to previous reported associations between narrower retinal arterioles and higher ACR [41–46] or urinary albumin excretion [47]. Similarly, consistent with the present analyses, most studies also found no association between venular calibre and ACR [37, 38, 41, 43, 44], although some reported associations between narrower venular calibre and higher urinary albumin excretion [47] and conversely, lower ACR [39, 40].

This study had several strengths in providing evidence of association between renal health and FD in a large well-characterised population-based study. Calculation of eGFR was undertaken using two methods: the CKD-EPI for SCr and the CKD-EPI equation for SCr and SCys [17]. The use of SCys offers improved sensitivity over eGFR based on SCr alone [48]. Moreover, although eGFR calculation based on the CKD-EPI equation for SCr is more commonly used in current clinical practice, a recent study using data from the UKBB has shown that eGFR calculated using the CKD-EPI equation for SCys better predicts the main problems associated with CKD (CVD, CVD-related mortality and renal failure) [49]. The UKBB dataset allowed us to compare associations using eGFR calculation based on eGFR and SCys. Additional analyses categorising participants by eGFR < 60 ml/min/1.73m^2^ based on the CKD-EPI equation for SCys showed no substantive difference to associations using the combined SCr and SCys equation (data not shown). UKBB was a well-characterised study that enabled consideration of a wide range of potential confounders representative of the UK population. For example, to account for the possibility that albuminuria and reduced renal function may represent manifestations of vascular dysfunction, sensitivity analyses were carried out with additional adjustment for covariates related to atherosclerotic vascular disease (specifically a history of stroke, heart attack and angina), although this failed to identify any additional significant findings beyond those reported in the current analyses. CRAE and CRVE are commonly estimated using microns as the unit of measurement. However, true quantification of calibre using fundus images is challenging due to differences in eye axial length, image magnification, accurate models of the optical path and due to a lack of accessible fiducial markers in the eye to facilitate calibration. Instead, software packages commonly use standardised approximations of optic disc diameter as a reference to convert pixels into microns, which leads to uncertainty in vessel calibre estimates. In contrast, VAMPIRE measures retinal microvascular calibre in pixels, reducing measurement error. Furthermore, statistical approaches based on standard deviation changes (Z scores) in RMP enable comparisons between studies that may vary by unit measures.

There were several limitations in our study. UKBB had lower than expected prevalence of several common diseases and may represent a “worried-well” within the UK and not entirely reflective of the population as a whole. In addition, ACR and eGFR were based on single measures of urinary albumin, urinary creatinine, and serum creatinine that differs from clinical definitions based on two measurements captured at least 3 months apart indicative of persistent albuminuria or reduced eGFR. Therefore, associations reported in this study may differ somewhat from those representative of clinical populations. Moreover, cases and controls were selected on the basis of ACR values and therefore secondary analyses between eGFR and RMP may not be entirely representative of the general population which may have higher levels of multiple morbidity. However, eGFR did not differ significantly between cases and controls, and key confounding influences (such as diabetes and blood pressure) were considered within adjusted models.

There was a high rate of image rejection in our study as a consequence of image quality including focus, contrast, positioning, eyelid and/or eye-lashes and excessive lighting (see Figure S1). Acquisition of UKBB vision data was time-limited and involved the collection of macula-centred fundus images only. Studies on the suitability of UKBB retinal images for semi-automated analysis have reported similar rejection rates [50, 51]. Software packages such as VAMPIRE provide improved sensitivity on optic disc centred images which capture a greater amount of the retinal microvasculature [52]. Furthermore, CRAE and CRVE are normally estimated using the six largest vessels, but macula-centred images typically exclude large vessels nasal to the optic disc. To facilitate measurement of RMPs in macula centred images, only those with at least four of the big six vessels, including the vessels of the retinal arcade (the largest retinal vessels) situated temporally to the optic disc, were included for analysis. As such, the absolute RMPs quantified as part of the current study may be limited in direct comparisons with those from other studies. Nevertheless, despite the high image rejection rate, the number of participants included in the study (920 cases and 970 controls) exceeded the numbers required to achieve statistical power.

Image rejection was slightly more common for older, non-white participants with a history of eye-surgery, higher LogMAR and lower IOP (see Table S1). Although significant, these differences are unlikely to influence the generalisability of our findings due to the small size of the differences detected in this large population. Mean age, for example, differed by 1 year, and a 3% difference in the use of blood pressure-lowering medication in cases was the largest difference in any categorical variable.

There are a range of factors that likely influence FD estimates and limit comparisons between studies and measurement algorithms. Variation in microvessel detection, measurement zones, automated segmentation protocols and thresholds used to create binary maps, may potentially confound associations between disease states and FD [34]. Moreover, FD values produced by various software packages may be sensitive to the use of different fundus cameras [34]. Although UKBB is a multicentre study, all images were captured using Topcon 3D OCT 1000 Mark 2 cameras.

Fundus images are routinely collected as part of diabetic eye screening and are a non-invasive imaging modality allowing the measurement of tissue changes associated with disease. The present study made use of semi-automated retinal vessel analysis. Recently a fully automated deep learning algorithm has been reported which classifies individuals as having CKD or no CKD based on macula-centred retinal images [53]. Other deep learning algorithms have been found to compare favourably to manual expert-led classification of images for conditions such as skin cancers [54] breast cancer [55] and diabetic retinopathy [56]. Using only fundus images as input, the performance of this algorithm compared favourably to CKD prediction equations based on traditional risk factors, and maintained similar performance in subgroups of participants with diabetes and hypertension [53]. Moreover, inclusion of addition of risk factors to the algorithm only marginally improved sensitivity and specificity [53]. The algorithm derived information from the entire fundus images rather than being limited to characteristics of the retinal vasculature but nevertheless made use of key features such as venular dilation, vessel rarefaction and changes associated with retinopathy [53]. This indicates some overlap with the present findings where vessel rarefaction (reduced FD) was associated with a greater risk of CKD.

## Conclusions

Overall, the findings from this case-control study of participants with healthy versus unhealthy ACR indicates that reduced retinal microvascular FD, i.e. sparser retinal microvascular networks, are associated with albuminuria and lower eGFR in a predominantly white population. No evidence was found for associations between ACR or eGFR and measures of retinal microvascular calibre and tortuosity.

## Supplementary Information

**Additional file 1: Figure S1**. Examples of images rejected during initial quality check. **Table S1**. Comparison of participant characteristics for those with and without imaging data.

## Data Availability

The UK Biobank resource is available to bona fide researchers for health-related research in the public interest. All researchers who wish to access the research resource must register with UK Biobank by completing the registration form in the Access Management System (AMS - https://bbams.ndph.ox.ac.uk/ams/).

## Abbreviations

ACR: Albumin to creatinine ratio
AVR: Arteriovenous ratio
CI: Confidence interval
CKD: Chronic kidney disease
CKD-EPI: Chronic Kidney Disease Epidemiology Collaboration
CRAE: Central retinal arteriolar equivalent
CRVE: Central retinal venular equivalent
CVD: Cardiovascular disease
Cys: Cystatin C
eGFR: Estimation of glomerular filtration rate
FD: Fractal dimension
FDa: Arteriolar fractal dimension
FDv: Venular fractal dimension
ICCs: Intra-class correlation coefficients
IOP: Intraocular pressure
logMAR: Logarithm of the minimal angle of resolution
OR: Odds ratio
RMP: Retinal microvascular parameters
Px: Pixels
SCr: Serum creatinine
SD: Standard deviation
Torta: Arteriolar tortuosity
Tortv: Venular tortuosity
UK: United Kingdom
UKBB: UK Biobank study
VAMPIRE: Vessel Assessment and Measurement Platform for Images of the REtina
