# Supplementary material for: Investigation of associations between retinal microvascular parameters and albuminuria in UK Biobank: a cross-sectional case-control study
Source: BMC Nephrol. 2021 Feb 25;22:72. doi: 10.1186/s12882-021-02273-6 (PMC7908698; doi:10.1186/s12882-021-02273-6)
Supplement: Supplementary file 1 — Additional file 1: Figure S1. Examples of images rejected during initial quality check. Table S1. Comparison of participant characteristics for those with and without imaging data. [file 12882_2021_2273_MOESM1_ESM.docx]

Figure S1. Examples of images rejected during initial quality check.


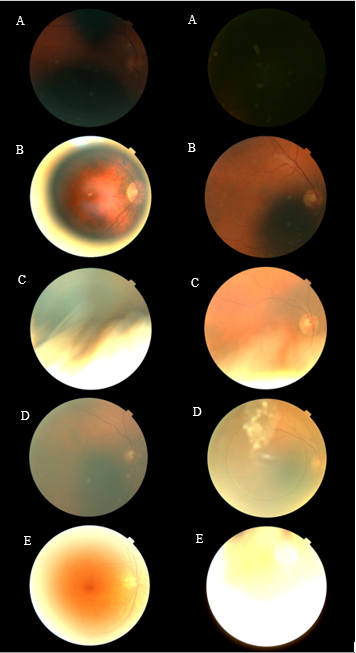


Figure S1. Examples of images rejected during initial quality check. A: dark images; B: images obscured by poor camera and light positioning; C: images obscured by eyelids and lashes; D: clouded images (N.B. contact lens visible in right image); E: images obscured by excessive lighting.

Table S1. Comparison of participant characteristics for those with and without imaging data.

|  |  | **RMP available** | **RMP not available** |  |
| --- | --- | --- | --- | --- |
|  |  | **Mean (SD)** | **Mean (SD)** | **p** |
| Age (years) |  | 58 (8) | 59 (8) | <0.001 |
| Waist circumference (cm) |  | 93 (15) | 94 (15) | 0.10 |
| Systolic blood pressure (mmHg) |  | 146 (22) | 147 (22) | 0.15 |
| eGFR (ml/min/1.73m^2^) |  | 88.8 (16.0) | 87.0 (16.4) | <0.001 |
| IOP (mmHg) |  | 16.48 (4.48) | 16.2 (4.33) | 0.02 |
| LogMAR |  | 0.03 (0.2) | 0.06 (0.22) | <0.001 |
|  |  |  |  |  |
|  |  | Number (%) | Number (%) | p |
| Sex (Female) |  | 1042 (55) | 2687 (53) | 0.06 |
| Blood pressure-lowering medication usage |  | 347 (18) | 1043 (21) | 0.049 |
| Diabetes mellitus |  | 209 (11) | 627 (12) | 0.16 |
| Ever smoked |  | 1104 (59) | 3069 (60) | 0.20 |
| White ethnicity |  | 1654 (88) | 4374 (86) | 0.04 |
| Ever consumed alcohol |  | 1764 (93) | 4752 (93) | 0.72 |
| Ever eye surgery |  | 127 (7) | 433 (9) | 0.02 |

Table S1. SD: standard deviation; eGFR: Estimated glomerular filtration rate; IOP: Intraocular pressure; LogMAR: visual acuity assessed using a Logarithm of the Minimum Angle of Resolution chart CRAE: central retina arteriolar equivalent; CRVE: central retinal venular equivalent; px: pixels; AVR: arteriovenous ratio; FDa/v: arteriolar/venular fractal dimension; Torta/v: arteriolar/venular tortuosity.
